# Functionalized Cellulose from Citrus Waste as a Sustainable Oil Adsorbent Material

**DOI:** 10.3390/polym18010082

**Published:** 2025-12-27

**Authors:** Loredana Maiuolo, Antonio Jiritano, Paola Costanzo, Federica Meringolo, Vincenzo Algieri, Giuseppe Arrabito, Giorgia Puleo, Antonio De Nino

**Affiliations:** 1 Department of Chemistry and Chemical Technologies, University of Calabria, Via P. Bucci, Cubo 12C, 87036 Rende, CS, Italy; loredana.maiuolo@unical.it (L.M.); antonio.jiritano@unical.it (A.J.); federica.meringolo@unical.it (F.M.); antonio.denino@unical.it (A.D.N.); 2 IRCCS NEUROMED—Istituto Neurologico Mediterraneo, Via Atinense 18, 86077 Pozzilli, IS, Italy; vincenzo.algieri@unical.it; 3 Department of Physics and Chemistry-Emilio Segrè, University of Palermo, 90128 Palermo, PA, Italy; giuseppedomenico.arrabito@unipa.it (G.A.); giorgia.puleo01@unipa.it (G.P.)

**Keywords:** cellulose, copolymerization, citrus waste, adsorption capacity

## Abstract

Oil spills are a significant environmental issue for marine wildlife and coastal communities. Cellulose derived from citrus peel industrial waste is an interesting, economical, and eco-friendly advantageous material that was used for the first time with the aim of developing a low-cost and sustainable sorbent for water purification. Citrus peel cellulose was grafted with methyl acrylate to enhance hydrophobicity and favor the oil adsorption in aqueous media. Grafting copolymerization was performed in a simple manner, and the conditions were optimized in terms of monomer concentration, amount of catalyst, time, and temperature. The modified cellulose polymer was obtained in different grafting percentages, with a maximum of 93% grafting. Fourier transform infrared spectroscopy (FTIR), ^1^H NMR, scanning electron microscopy (SEM), and electrochemical impedance spectroscopy (EIS) analysis were used to confirm the graft copolymerization of poly(methyl acrylate) (PMA) onto the mercerized cellulose. Finally, the oil adsorption capacity of selected copolymers from freshwater, artificial seawater, and seawater samples was tested in a continuous-flow system. The results showed promising performance retaining diesel in seawater (4.01 g oil/g cellulose), demonstrating the use of agri-food waste as a natural sorbent in oil removal.

## 1. Introduction

Cellulose is the most abundant biopolymer in the world, with many advantageous properties, including biodegradability, biocompatibility, and renewability. The plant-based materials, for, e.g., coir and cotton, present an amount of cellulose ranging from 31 to 96%, respectively [1]. For these reasons, numerous extraction processes have been developed in recent years to obtain materials suitable for a wide range of industrial [2] and advanced applications [3,4], including pharmaceutical uses, membrane and filtration systems, and drug delivery, eventually extending their application to the field of stimuli-responsive microrobots [5]. Moreover, cellulose can also be recovered from a variety of sources, including wood-based biomass, agricultural, and industrial wastes. Agri-food waste was largely investigated in recent years as a raw material for developing efficient, cheaper, and renewable adsorbents for water pollution remediation [6]. They can be derived from crop straw and forest residues and are mainly composed of lignocellulosic materials with a high content of cellulose [7]. Among these raw materials, citrus peel is a rich source of cellulose, and different chemical routes were developed for its extraction from citrus processing wastes (CPWs) [8,9,10]. CPWs obtained from orange and lemon juice industrial production are traditionally associated with the production of pectin on an industrial scale, but a growing interest in cellulose derived from waste orange peel is observed [11,12,13]. The “best out-of-waste” is the more appropriate definition for the valorization of cellulose derived from CPWs, as a result of the global push on the circular economy. Although cellulose is insoluble in water and in the most common organic solvents, the large number of polar hydroxyl groups allows for easy modification of the cellulose surface to fine-tune its chemical and mechanical properties for advanced applications [14]. Grafting polymerization is a common and effective method for cellulose modification, involving the covalent bonding of a small polymer side chain to form a copolymer with branched structures. This kind of functionalization not only improves compatibility with different types of matrices in the formation of composites, imparts flame retardant properties, and enhances the moisture resistance [15] but also increases hydrophobicity and enables oil absorption [16,17,18,19]. In particular, the ability to absorb oil with an abundant and low-cost material, like cellulose, was extensively investigated in recent years [20], since oil spills are considered one of the most dangerous and hazardous contaminants for water, especially for marine ecosystems. In fact, marine oil spills occur frequently, causing great damage to the environment [21]. Developing an effective purification method has therefore become a significant environmental challenge. Among various remediation techniques, adsorption has proven to be one of the most efficient and cost-effective approaches for water purification [22,23]. In this context, cellulose-based materials represent a promising option for designing effective adsorbents [24].

In light of our experience with chemical modifications of cellulose [25,26] and the preparation of adsorbent materials for water purification [27,28], we hypothesized to chemically modify cellulose in order to obtain a more hydrophobic material for this purpose. In this work, we employed a cellulose derived from CPWs generated during pectin extraction—previously modified successfully in our recent work [29]—as a starting material to produce an effective oil sorbent. Graft copolymerization reactions with methyl acrylate were investigated by exploring different chemical conditions, and as a result, a simple on-air reaction was realized to afford new materials with different grafting percentages. The choice of poly(methyl acrylate) (PMA) as a grafting agent for cellulose is based on the known biocompatibility of this polymer [30]. The final materials were chemically characterized by Fourier transform infrared (FT-IR) spectroscopy and ^1^H NMR, and their morphologies were analyzed by a scanning electron microscope (SEM). Contact angle measurement and electrochemical impedance spectroscopy (EIS) analysis were also conducted on selected materials to evaluate changes in interfacial properties induced by grafting. Fuel sorption capacity of the selected materials was evaluated in both batch and continuous flow, demonstrating an improvement compared to unmodified cellulose and confirming the usability of this procedure for producing a new type of bio-based adsorbent material.

## 2. Materials and Methods

### 2.1. Chemicals

Cellulose extracted from citrus peel waste was provided by JRS Silvateam Ingredients S.r.l. (Rende, CS, Italy). Microcrystalline cellulose type 102 was purchased from Roquette (Lestrem, France) at high purity. Sodium hydroxide, acetic acid, potassium persulfate (KPS), ferrous ammonium sulfate (FAS), and acetone were purchased from Sigma Aldrich (St. Louis, MO, USA) at analytical grade and used without further purification. Methyl acrylate was purchased from Sigma Aldrich (St. Louis, MO, USA) at 99% purity grade and used after distillation. Gasoline oil (density at 15 °C = 0.645–0.665 g/cm^3^, kinematic viscosity = 0.45 cSt at 20 °C) and diesel oil (density at 15 °C = 0.835 g/cm^3^, kinematic viscosity = 3.0 cSt at 20 °C) collected from the local service station were used as experimental oils.

### 2.2. Mercerization of Cellulose

In a round-bottom flask, 5 g of cellulose (MCC or Citrus peel wastes) and 150 mL of 2% NaOH solution were placed, and the mixture was stirred at room temperature for 3 h. After this time, the solid was filtered through a Buchner funnel and washed, first with distilled water until the pH was neutral, then with a 1% acetic acid solution (to protonate the residual cellulose-O-Na groups into cellulose-O-H ones), and finally with distilled water again until pH 7. The solid was dried slowly, first at room temperature overnight and then in an oven, gradually increasing the temperature by almost 10 °C per hour until a temperature of 70 °C was reached, while breaking up the larger aggregates, thus obtaining fine solid particles. Finally, the mercerized cellulose was ground in a mortar and sieved to make the solid particles as homogeneous and small as possible.

### 2.3. Graft Copolymerization of Cellulose

In total, 0.5 g of mercerized cellulose (dried overnight in an oven at a temperature of 60–70 °C) and 100 mL of distilled water were placed in a two-necked round-bottom flask equipped with a magnetic stirrer and a reflux condenser and left under slow stirring for 24 h (soaking step) at room temperature. Then, the mixture was heated to 40 °C, and known amounts of KPS and FAS were added, leaving the mixture under stirring for 10–15 min. Subsequently, methyl acrylate was slowly added, and the reaction was left under stirring at a fixed temperature for the desired time. After this time, the stirring was stopped, and the system was allowed to cool. The solid was then filtered through a Buchner funnel and washed with distilled water until the pH was 7. Subsequently, the material was washed in a Soxhlet apparatus for 72 h using acetone as solvent to remove the homopolymer formed during the reaction. The obtained material was dried in an oven at 60–70 °C overnight and finally weighed. The percentage grafting (P_G_) was calculated as

(1) $$\mathrm{Percent}\;\mathrm{grafting}\;(P_{G})=\frac{W_{g}-W}{W_{g}}\times 100$$

where *W_g_* is the weight of grafted cellulose after homopolymer extraction, and *W* is the weight of the starting material.

### 2.4. Sorption Capacity Test in Continuous Flow Using Fuel/Water System

The fuel removal assays were performed using two procedures, in both cases using fresh, substitute seawater and real seawater. The first procedure for fuel removal, specifically gasoline and diesel, was performed by using a peristaltic pump, connected to a cartridge in which the solution was passed continuously in up-flow mode, and the solution was collected at the end of the system. The sorbent selected materials, approximately 20 mg, were placed inside the cartridge. The peristaltic pump was used at a flow rate of 4 mL/min, the column had a 4 mm × 7.5 cm internal diameter/length, and the packing density of the adsorbent was 0.8 g/cm^3^. The fuel was recovered by centrifugation, putting the cartridge directly inside a centrifuge tube (VivaspinTM 6 centrifugal concentrator tubes were purchased from Fischer Scientific, Waltham, MA, USA) to collect the oil. The oil adsorption capacity S, expressed in g/g, was calculated using the following formula:

(2) $$S\;=\;\frac{M_{t}\;-\;M_{0}}{S_{0}}$$

where *M*_0_ is the weight of the reaction flask, *M_t_* is the weight of the reaction flask plus the collected oil after the process, and *S*_0_ is the initial weight of the sorbent.

In this system, the artificial seawater was prepared according to ASTM D1141-98 [31], and a real sample of seawater was taken from the Ionian Sea. The employed apparatus is the same as that reported in a previous work [28]. The procedure was carried out in triplicate, and the mean of the results was reported.

### 2.5. Sorption Capacity Test in Batch Using Fuel/Water System

The test in batch was performed in accordance with the Standard Test Method for Sorbent Performance of Adsorbents (ASTM F726-99) [32]. A volume of 50 mL of water and a different amount of fuel (from 50 to 500 mg) were added to a 100 mL conical glass flask and put under stirring. After that, approximately 20 mg of the modified cellulose material WC20 was placed inside the conical glass flask. After a certain amount of time (from 5 to 300 s), the adsorbent was recovered and centrifuged to recover the adsorbed fuel: the material was placed in a centrifugal concentrator tube to collect the oil. The adsorbed materials were centrifuged at 500 rpm with a rotor radius of 11 cm for 5 min until the oil was separated at the bottom. The oil adsorption capacity S, expressed in g/g, was calculated with the previous Equation (2). The procedure was carried out in triplicate, and the mean of the results was reported.

### 2.6. Fourier Transform Infrared (FT-IR) Analysis

FT-IR spectra (KBr pellets) of selected materials (MCC, WC, MWC, and WC5) were recorded with a Jasco FT/IR-4200 spectrometer (JASCO EUROPE s.r.l.—Cremella, LC, Italy) in a frequency range between 450 and 4000 cm^−1^, with a resolution of 2 cm^−1^.

### 2.7. Scanning Electron Microscope (SEM) Analysis

About 20 mg of the oven-dried sample was charged on pin stub. The SEM machine (Thermo Fisher Scientific—Phenom-World B.V., Eindhoven, The Netherlands) was allowed to stabilize for 120 s before setting the parameters to be used. Imaging of the sample was performed at 15 kV, with pressure at 0.003 Pa, and set at 1000 magnification.

### 2.8. NMR Characterization of WC20 and PMA

The ^1^H NMR experiment for WC20 was recorded at 500 MHz, in DMSO-d_6_ as solvent, using tetramethylsilane (TMS) as an internal standard and at 37 °C after dilution of the sample at 50 °C in DMSO-d_6_ (Bruker Avance 500 MHz with a 5 mm TBO probe, Rheinstetten, Germany). The experiment was realized in water suppression at 3.12 ppm. Chemical shifts are given in parts per million and coupling constants in Hertz.

### 2.9. Contact Angle Measurements

Several pellets of WC20 were prepared by the following pressing procedure: almost 70 mg of WC20 was charged into an 80-ton hydraulic tablet press.

The surface wettability of the pellet was evaluated by static contact angle measurements carried out at room temperature using a CAM 200 device (KSV Instruments, Ltd., Helsinki, Finland). On the surface of each pellet, a 5 µL drop of gasoline or diesel was deposited by a micro-syringe. For comparison purposes, water was also used as a testing liquid. The angle between the surface tangent on the liquid–air interface and the tangent on the membrane–liquid interface at their intersection was measured from 5 video-captured images taken at 20 ms intervals immediately after drop deposition.

### 2.10. Electrochemical Impedance Spectroscopy Analysis (EIS)

The interfacial electrical properties of the functionalized cellulose suspensions in water were investigated by EIS. In particular, this analysis permits us to analyze the dielectric response at the particle–water interface, allowing the assessment of how chemical grafting influences the interfacial polarization and ionic mobility in the aqueous medium [33,34], and ultimately finding a correlation to the fuel removal capacity.

EIS measurements were performed on aqueous suspensions of the selected materials at concentrations of 1 mg/mL and 10 mg/mL to investigate how both composition and concentration influence the electrical response of the system and to obtain information on their surface properties. To this aim, a miniaturized serigraphic three-electrode electrochemical sensor deposited on a polyester substrate (ItalSens Carbon SPE, PalmSens BV Vleugelboot 22, 3991 CL Houten, The Netherlands) was used. The device was connected to an AUTOlab potentiostat PGSTAT 128 N (Woudwetering 3-5, 3543 AV Utrecht, The Netherlands) controlled via NOVA 2.1.5 software. The sensing platform comprised a circular graphite working electrode with a 3 mm diameter, a Ag pseudo-reference electrode, and a graphite counter electrode. All experiments were carried out in pure water, without the addition of supporting electrolytes. Measurements were acquired over a frequency range from 100 kHz to 0.1 Hz, using ten frequency points per decade. The excitation signal consisted of a sinusoidal perturbation of 5 mV (rms) amplitude, with a total acquisition time of approximately 200 s per spectrum. All measurements were performed in triplicate. The experimental error was calculated as the percentage error associated with the polarization resistance Rp values, obtained by fitting the experimental data using the NOVA 2.1.5 software.

## 3. Results and Discussion

### 3.1. Graft Copolymerization onto Cellulose Fibers Derived from Citrus Peel Wastes

In this study, the preparation of modified cellulose via the graft copolymerization method includes the mercerization pretreatment step. The natural fiber derived from CPWs was pre-treated with a 2% sodium hydroxide solution (mercerization) to increase its reactivity. In this process, Cellulose I was converted to Cellulose II (more reactive and thermodynamically stable): Na^+^ cations penetrate the intercrystalline spaces, causing the cellulose to swell. Consequently, the interchain hydrogen bonds are broken. After washing, neutralization, and drying, the cellulose undergoes an irreversible morphological and structural change into Cellulose II [35]. Mercerization also allows for the purification of the fiber, which is obtained from agri-food waste (citrus peels) and contains various substances, some of which are susceptible to treatment with a basic solution (including lignin and hemicellulose) [36].

**Scheme 1:**
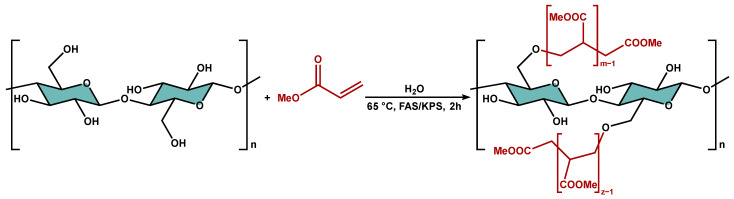
Grafting of mercerized cellulose with methyl acrylate by radical polymerization.

A radical graft was then performed on the mercerized and non-mercerized fibers using methyl acrylate as a monomer (Scheme 1) in order to increase the lipophilic component through the grafting of an acrylic polymer.

The use of ferrous ammonium sulfate–potassium persulfate (FAS–KPS) as redox initiator was extensively reported as an efficient method for radical polymerization of cellulose materials [37,38].

As a preliminary study, we tried to compare the effect of functionalization on microcrystalline cellulose (MCC) and cellulose derived from the CPWs (WC), by using a slightly modified method reported in the literature for natural fibers [39]. In this procedure, the reaction was conducted at 80 °C for 24 h, with a ratio of FAS/KPS of 1:180. In Table 1, the results of grafting copolymerization with methyl acrylate under an inert atmosphere and in degassed water, to avoid the presence of O_2_, are reported.

As shown in Table 1, the best result was obtained on mercerized WC, although the P_G_ was only at 16%. In this reaction condition, a high degree of omo-polymerization of the monomer was measured (almost 88.2%). These preliminary results confirmed the mercerization process as a crucial step towards the cellulose reactivity (entries 2 and 4, Table 1) and that the WC is more responsive than MCC to the functionalization procedure (entries 3 and 4, Table 1). In light of these results, we decided to investigate the mercerized WC (MWC) as a promising starting material for the production of lipophilic material for adsorbent purposes.

Then, the optimization of the grafting reaction on the MWC was carried out by keeping the concentration of initiator and cross-linker constant, and the other parameters, such as time, temperature, and monomer to fiber ratio, were varied.

Table 2 reports the different values of graft yields obtained during optimization of the reaction parameters. For all the materials, an extensive final washing step with acetone (a good solvent for both the omo-polymer and the unreacted monomers) resulted in a grafted cellulose, free of unbonded PMA or methyl acrylate. It is worth noting that we did not measure significant weight loss when we performed a second washing work-up procedure (data not reported).

Firstly, we tried to simplify the preliminarily tested grafting procedure reported in Table 1, entry 4, by working in an open-air system and avoiding the use of a nitrogen atmosphere.

**Figure 1:**
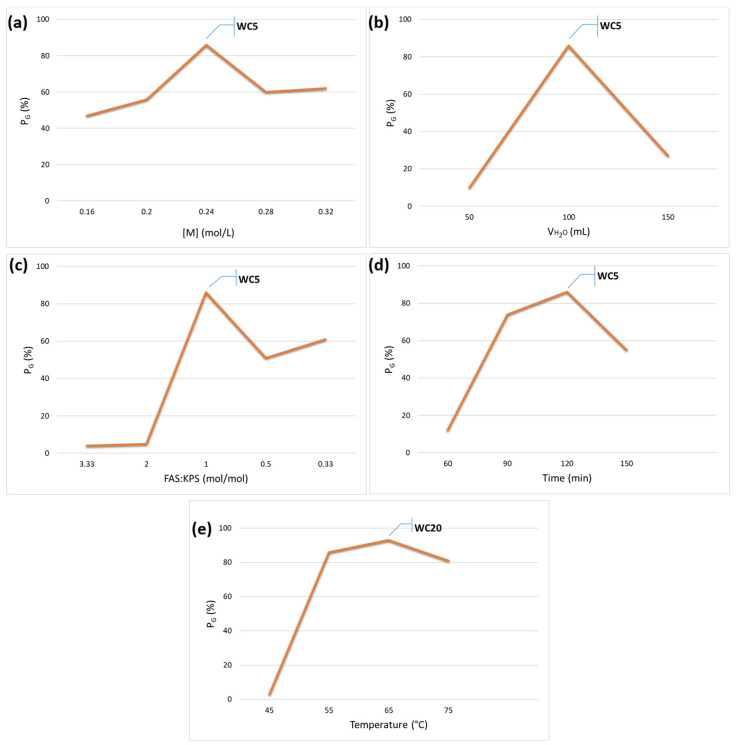
Optimization of graft copolymerization of methyl acrylate onto MWC for (**a**) monomer; (**b**) solvent amount; (**c**) initiator ratio; (**d**) reaction time; (**e**) temperature.

In Figure 1, all the graphs relating to the optimized parameters are summarized.

We started by optimizing the monomer concentration (entries 1–5, Table 2). As the concentration of methyl acrylate increases, the number of monomeric radicals available for propagation also rises, leading to a higher P_G_ (entry 3, Table 2). Beyond this concentration, however, the excess monomer radicals predominantly participate in termination reactions, thereby causing a subsequent decrease in the P_G_ (Figure 1a). The reaction was repeated under an inert atmosphere at the optimal monomer concentration, but no improvement was observed (entry 6, Table 2). This result can be explained by the fact that, although molecular oxygen reacts with polymer radicals to form less reactive peroxyl radicals, these radicals can still take hydrogen atoms from the polymer, creating hydroperoxides and new polymer radicals. The newly formed radicals can then react with methyl acrylate monomers, thereby promoting further polymerization [40]. This mechanism accounts for the higher P_G_ observed under oxygenated conditions (entry 3, Table 2) compared to that obtained in an inert atmosphere (entry 6, Table 2). In contrast, when the reaction was carried out without the 24 h water soaking step, the P_G_ drastically decreased to 2% (entry 7, Table 2), demonstrating that the swelling phase of the material in the reaction solvent is crucial for exposing the active sites. This observation was confirmed when the reaction volume was reduced by half (entry 8, Table 2). Therefore, the optimal solvent condition was achieved using 100 mL of water as the reaction medium (Figure 1b). The subsequent decrease in P_G_ beyond this optimal volume can be attributed to excessive solvation of the monomer, which limits its accessibility to the active sites [41]. Additionally, this decline may be associated with the dilution effect caused by increasing the amount of water, which effectively reduces the “concentration” of active sites on the fiber surface and consequently hinders graft growth (entry 9, Table 2). The effect of the FAS–KPS molar ratio was also investigated (entries 10–13, Table 2). The previously established 1:1 ratio between the catalyst and initiator yielded the highest P_G_. As expected, reducing the amount of KPS resulted in negligible grafting (entries 10–11, Table 2), whereas decreasing the amount of FAS (entries 12–13, Table 2) still allowed the reaction to proceed, since KPS serves as the radical source while FAS functions as the catalyst for radical formation (Figure 1c). The effect of reaction time was investigated over a range of 60 to 150 min (entries 14–16, Table 2). With an increasing reaction time, chain propagation is enhanced, leading to a corresponding increase in the P_G_ up to an optimal duration at which the grafting degree reaches its maximum at 120 min of reaction (entry 3, Table 2). However, beyond this optimal time, the reaction medium becomes increasingly viscous, hindering monomer diffusion (entry 16, Table 2). As a result, termination reactions become predominant, leading to a decline in grafting efficiency (Figure 1d). Finally, the effect of the temperature was evaluated. At temperatures below 45 °C (entry 17, Table 2), grafting does not occur because the initiator does not decompose, and consequently, radical species are not formed. Increasing the temperature enhances the diffusion of persulfate and monomer (Figure 1e), resulting in an increase in the P_G_ to 93% at 65 °C (entry 18, Table 2). A further increase in temperature (T = 75 °C) makes the initiator less effective due to a shorter half-life, and consequently, the grafting process becomes less effective (entry 19, Table 2), as already described in the literature [39].

The optimum conditions for maximum graft yield (93%) as given in Table 2, entry 19, were monomer—(0.24 mol/L); solvent—100 mL; FAS/KPS initiator (1:1); time—120 min; temperature—65 °C.

### 3.2. Characterization of Cellulose Copolymerized with Methyl Acrylate

#### 3.2.1. Fourier Transform Infrared (FT-IR)

Both the native fiber, the mercerized native fiber, and all grafted materials were characterized by FT-IR spectroscopy and scanning electron microscopy (SEM).

**Figure 2:**
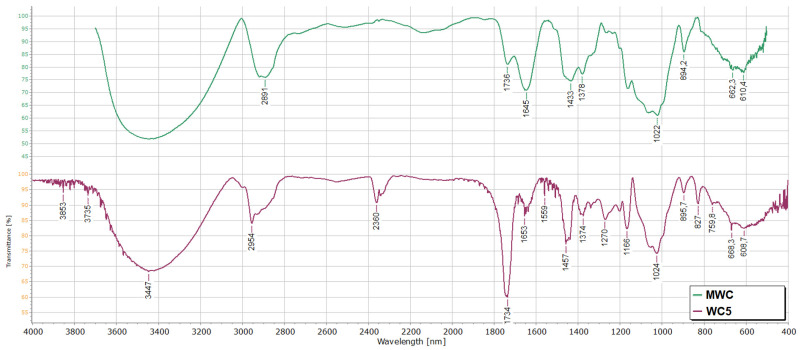
FTIR spectra of cellulose derived from CPWs after mercerization (top curve in green) and WC5; the material at a high degree of functionalization (bottom curve in purple).

From the analysis of the acquired FT-IR spectra, we can draw the following conclusions:

(1) Both the MCC and the natural fiber spectra contain common signals due to the functional groups present in cellulose. The only significant peak that differentiates the two spectra is the one at 1740 cm^−1^, present exclusively in the spectrum of the natural fiber, and which is attributable to the C=O stretching of the hemicellulose (See Figure S1 in the Supplementary Materials).
(2) The enhanced peak intensity near 894 cm^−1^, in the spectrum of MWC, indicates an increased amorphous content [42], resulting in greater hydroxyl group accessibility. Consequently, the mercerized fiber exhibits higher reactivity than the untreated cellulose, as grafting does not occur on the raw fiber. Furthermore, the peak at 1740 cm^−1^ is less intense than the native cellulose, suggesting that the pretreatment with NaOH solution helped to remove the hemicellulose components (see Figure S2 in the Supplementary Materials).
(3) At higher P_G_ (material WC5), the intensity of the peak at 1734 cm^−1^ increases markedly, reflecting a substantial rise in C=O ester group content (Figure 2). Additionally, grafting with PMA induces the appearance of a peak at 2954 cm^−1^, probably due to the stretching of CH_2_ and CH_3_ groups, and the presence of a weak band at 827 cm^−1^, likely associated with the CH_2_ group rocking vibrations of the grafted chains [43].

#### 3.2.2. Morphological Analysis by Scanning Electron Microscopy (SEM)

**Figure 3:**
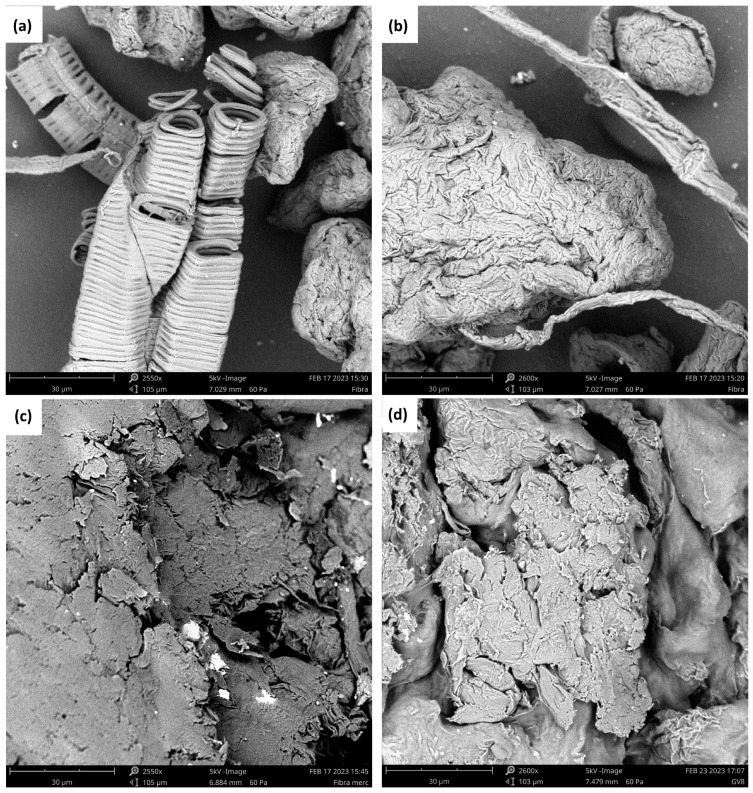
SEM images of (**a**,**b**) native cellulose derived from CPWs, (**c**) mercerized cellulose, MWC, and (**d**) cellulose grafted with PMA, WC5.

The materials were also examined by SEM to detect the differences in surface morphologies between the natural fiber (Figure 3a,b), the mercerized fiber (Figure 3c), and the functionalized fiber (Figure 3d). From the SEM images, it was possible to detect particular three-dimensional helical structures present exclusively in the starting natural fiber (Figure 3a). After mercerization, these structures are no longer present, probably because they are due to substances that are degraded or removed during the alkaline treatment, such as hemicellulose [36]. Furthermore, the surface of the mercerized fiber appears smoother than that of the rough starting fiber (Figure 3b,c). Finally, it can be observed that after grafting, the fiber surface appears rough again; this increase in roughness is attributed to a high grafting density (Figure 3d).

#### 3.2.3. NMR Characterization of WC20 and PMA

**Figure 4:**
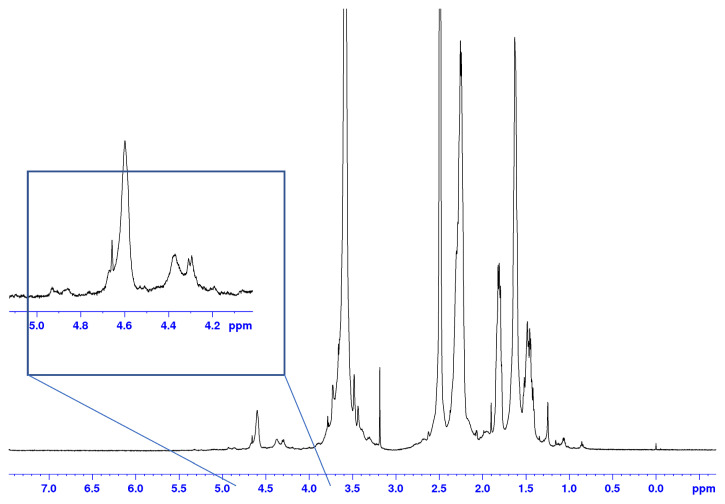
^1^H NMR spectra (in DMSO-d_6_) of material WC20 recorded at 37 °C.

^1^H NMR of material WC20 was performed to confirm the chemical structure, as reported in Figure 4.

The spectrum shows the signals inherent to cellulose and PMA. The peaks between 4.96 and 4.08 ppm (as shown in the magnification) are attributed to some protons of the cellulose backbone and are similar to previous reports on modified cellulose-based materials [44,45,46], while the PMA signals are assigned by acquired ^1^H NMR and the literature data [47]. In particular, all PMA signals are slightly moved towards higher fields compared to those of PMA alone (see Figure S11, in Supplementary Materials).

#### 3.2.4. Contact Angle Measurements

We tried to test the affinity of the material WC20 towards gasoline and diesel by contact angle measurements. Unfortunately, in the case of gasoline and diesel, the pellet immediately adsorbed the drop, and it was not possible to determine the contact angle (Figure S12, in Supplementary Materials). We noted, in the case of gasoline, a faster adsorption compared to diesel, probably due to the higher viscosity of the second testing liquid.

The experiment was also performed for water, and it was possible to correctly measure the contact angle, which was 66.8 ± 2° (Figure S13, Supplementary Materials).

### 3.3. Adsorption Capacity Using Different Oil Phases

The continuous-flow system was used to test different materials for their sorption capacity toward diesel and gasoline. The materials used in this study included microcrystalline cellulose (MCC), mercerized WC (MWC), and various WCs functionalized with different P_G_: WC11, WC4, and WC20, corresponding to 27%, 56%, and 93% grafting, respectively. These samples were analyzed to evaluate the relationship between the percentage of grafting (P_G_) and the measured sorption capacity. Table 3 reports the sorption capacity values, S (g/g), calculated using the previously described Equation (3) for the selected materials.

As shown in Table 3, the adsorption capacity is directly correlated with the P_G_ value of the sample. Specifically, the sample with the highest P_G_, WC20, also exhibited the greatest adsorption capacity for both diesel and gasoline. Diesel was adsorbed more effectively than gasoline, with adsorption capacities of 3.87 g/g and 2.79 g/g, respectively (entries 2 and 3, Table 3). Then, to evaluate the influence of the salts diluted in water on the affinity between the cellulose material and the oil, the aqueous phase was substituted with artificial seawater. As expected, a slight improvement in the adsorption capacity was observed when working in these simulated conditions (entries 4 and 5, Table 3). Encouraged by these results, we used a real sample of seawater from the Ionian Sea in Italy to test the functionalized celluloses in a more complex system. As a promising outcome, the WC20 sample exhibited the highest adsorption capacity for removing diesel from seawater (entry 6, Table 3), achieving an S value of 4.01 g/g. In the case of gasoline, the maximum adsorption capacity measured was 2.95 g/g. It is worth noting that the graft copolymerization of methyl acrylate onto the cellulose surface played a key role in enhancing the adsorption capacity of pure cellulose. As shown in Table 3, MCC exhibited an S_0_ value seven times lower than that of WC20 for diesel and six times lower for gasoline (entries 2 and 3). Furthermore, as reported in Table 3, the mercerization of cellulose enhances the hydrophilicity of the cellulose materials, since MWC showed no ability to adsorb fuels. Moreover, a too-low P_G_ value did not allow the material to recover a partial adsorption capacity, as measured for WC11. Different waste-derived cellulose materials were reported in the literature for oil adsorption, such as nanocellulose derived from kapok fibers for the adsorption of mineral or vegetable oil [48]; banana fibers for the adsorption of a high-density oil [17], or oil palm empty fruit bunch or Cocoa pods for the adsorption of crude oil [49]. To the best of our knowledge, only a few examples of cellulose derived from agri-food waste were used after a simple modification step for fuel adsorption. The best result reported in the literature was for cellulose fibers derived from corn straw, which adsorbed up to 52 g/g of diesel, although over 7 days; however, the material consists of macroscopic fibers rather than a powder [50]. Instead, cellulose derived from Pomelo peels and modified with acetic anhydride or styrene exhibited an adsorption capacity for diesel of 16.4 g/g and 18.9 g/g, respectively [51]. In many cases, the cellulose material was converted to aerogel before being further chemically modified or coupled with other materials to obtain composites [52]. In these cases, the high porosity and high specific surface area allow for the adsorption of amounts of diesel ranging from 12 g/g of a functionalized aerogel obtained from rice straw [53] to 83 g/g of a modified aerogel coming from bamboo powder [54].

Although the oil sorption capacity of our material seems lower, if compared to these materials, we achieved the aim of adding value to a waste in a relatively simple manner. In detail, the lower sorption capacity could be due to the impossibility to maintain packing continuity, leading to limited contact time between the filtering material and the fluids. Noteworthy is that, for the first time, our waste-derived material has been shown to be usable in a continuous-flow system and for an application with real seawater.

#### 3.3.1. Sorption Kinetics of WC20 in Diesel/Water and Gasoline/Water Systems

The sorption kinetics were evaluated for the best-performing material, WC20. The obtained results were fitted using two commonly applied models to describe this kind of study, the pseudo-first-order and the pseudo-second-order model, using, for both models, the linear equations reported as follows:

(3) $$\mathrm{Pseudo}\text{-}\mathrm{first}\text{-}\mathrm{order}\;=\;\ln\left(q_{e}-q_{t}\right)=\ln q_{e}-k_{1}t$$

(4) $$\mathrm{Pseudo}\text{-}\mathrm{second}\text{-}\mathrm{order}=\frac{t}{q_{t}}=\frac{1}{k_{2}q_{e}^{2}}+\frac{1}{q_{e}}t$$

where q_e_ is the maximum fuel sorbed at the equilibrium, q_t_ is the fuel sorbed at time t, and k_1_ and k_2_ are the first- and second-order rate constants. We found that the equilibrium was reached at 2 min for both diesel and gasoline. The following table reports the values of the kinetic parameters for both the fuels and the experimental and theoretical values of q_e_.

We employed pseudo-first-order and pseudo-second-order kinetic models to elucidate the overall sorption mechanism. The pseudo-first-order model typically describes systems characterized by a linear sorption equilibrium, a predominant dependence on time rather than solute concentration, and a rapid approach to equilibrium—phenomena that generally occur during the early stage of the sorption process. Conversely, the pseudo-second-order model is more appropriate for systems in which chemical interactions constitute the predominant driving force compared with purely physical phenomena such as occlusion, inclusion, or simple diffusion.

**Figure 5:**
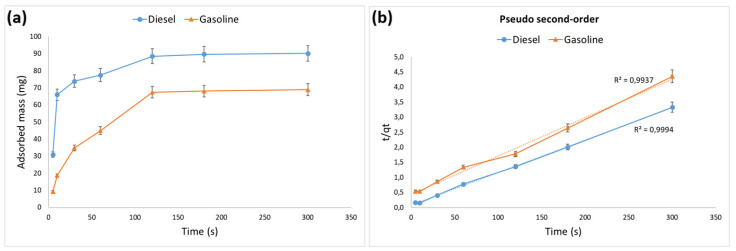
Graphs for gasoline and diesel kinetics (**a**) and pseudo-second-order (**b**) sorption linear fitting of WC20 in gasoline and diesel/water systems.

Figure 5 reports the kinetic profiles for WC20 following the pseudo-second-order models. Analysis of the correlation coefficients (Table 4) demonstrates that the pseudo-second-order model provides the best fit for the experimental data in both gasoline/water and diesel/water biphasic systems. The pseudo-first-order model graphs for WC20 were reported in the Supplementary Materials (Figures S3 and S4).

Since the rate-limiting step in the pseudo-second-order kinetic model is typically attributed to chemisorption occurring at the surface, the results strongly suggest that chemical interactions govern the sorption mechanism in the present study. Moreover, WC20 exhibits a slightly higher affinity for the diesel phase compared to gasoline.

#### 3.3.2. Adsorption Isotherms of WC20 in Diesel/Water and Gasoline/Water Systems

We also investigated which of the Langmuir or Freundlich isotherm models best represented the experimental data in our system. The Langmuir isotherm is a model relating to a type of process that consists of a monolayer adsorption on a homogeneous surface where the functional groups of the adsorbent are equally distributed. The equation relative to the first model is the following:

(5) $$q_{e}=q_{max}\frac{K_{L}C_{e}}{1+K_{L}C_{e}}$$

in which q_max_ is the maximum sorption capacity (g/g), q_e_ is the quantity of oil sorbed at the equilibrium (g/g), C_e_ is the oil concentration at the equilibrium (g/L), and K_L_ is the Langmuir isotherm constant (L/g). The isotherm graphs for WC20 related to the initial oil concentration were reported in the Supplementary Materials (see Figures S5 and S6). Another important parameter is the dimensionless separation factor (R_L_), commonly used because it can be calculated by using the Langmuir parameter K_L_ [55]. The equation is the following:

(6) $$R_{L}=\frac{1}{1+K_{L}C_{0}}$$

in which K_L_ (L/g) is the Langmuir constant, and C_0_ is the initial oil concentration (g/L). This value is important because it estimates whether the process is unfavorable (R_L_ > 1), linear (R_L_ = 1), favorable (0 < R_L_ < 1), or irreversible (R_L_ = 0).

**Figure 6:**
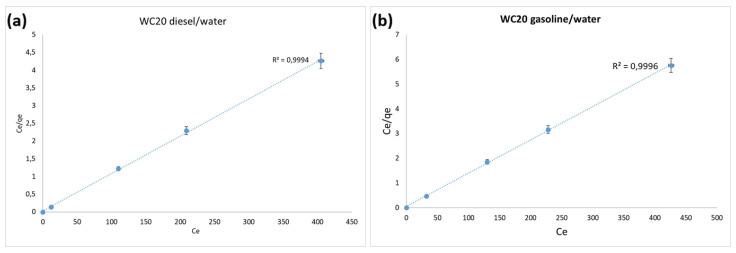
Langmuir plots for WC20 in diesel (plot (**a**)) and gasoline (plot (**b**)).

The other isotherm model applied was the Freundlich model, which describes non-ideal adsorption on a heterogeneous surface, where the adsorption process occurs in multiple layers rather than a single monolayer. It is expressed by the following equation:

(7) $$q_{e}=K_{F}C_{e}^{1/n}$$

in which K_F_ represents the Freundlich equilibrium constant related to adsorption capacity, n represents the affinity of the fuel for the sorbent material, and q_e_ is the unit weight of fuel sorbed at the equilibrium for the unit weight of the sorbent material. The best plots relative to the Langmuir model are reported in Figure 6.

The isotherm plots shown in Figure 6, together with the corresponding correlation coefficients (R^2^ values) reported in Table 4, clearly indicate that the Langmuir model provides the most appropriate description of the experimental data. The Langmuir isotherm assumes monolayer adsorption onto a surface bearing a finite number of energetically equivalent active sites arranged homogeneously. In this system, the sorption mechanism is governed not only by surface interactions but also by capillary phenomena, which further contribute to the overall uptake process.

By contrast, the Freundlich model yielded lower correlation coefficients, supporting its typical suitability for heterogeneous or multilayer adsorption processes rather than the uniform surface interactions observed here (see Figures S7 and S8 in the Supplementary Materials). The fitted parameters for both models are reported in Table 5.

### 3.4. Results of the Electrochemical Impedance Spectroscopy (EIS) Analysis

To elucidate how the particle functionalization and suspension concentration influence the electrical response of the system, EIS measurements were conducted using a miniaturized three-electrode platform.

Cellulose is an electrical insulator, characterized by high resistance (>108 Ohms). Similarly, ultrapure water is characterized by very low conductivity (typically 0.055 µS/cm) because of the low ion concentrations that result from the self-dissociation of water. Notably, cellulose surface functionalization is a key factor to improve cellulose dispersion stability [56]. EIS measurements were performed without a supporting electrolyte, permitting direct probing of the intrinsic interfacial polarization arising from the interfaces between cellule particles and grafted acrylate chains. In fact, the presence of dissolved ions in the water phase could cause their accumulation at the cellulose/water interface, thus screening the surface polarization due to the acrylate chains grafting. This approach enables the direct observation of charge accumulation and redistribution at the particle interfaces, consistent with the Maxwell–Wagner–Sillars (MWS) framework for solid composite systems [57].

**Figure 7:**
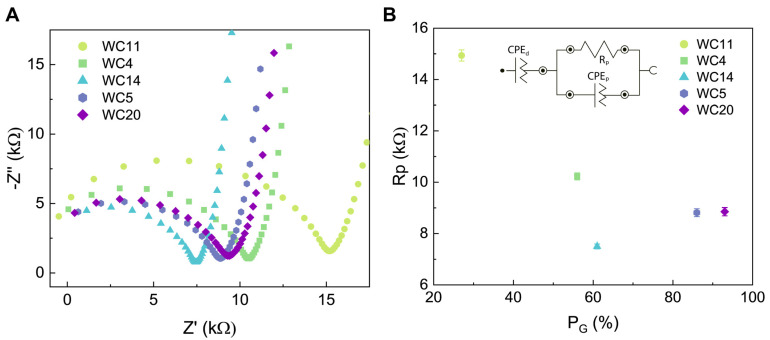
(**A**) Nyquist plots of aqueous suspensions (1 mg/mL) of WC4, WC5, WC11, WC14, and WC20. (**B**) Graph showing polarization resistance (Rp) as a function of grafting percentage (P_G_) in the samples under investigation. The inset illustrates the equivalent circuit used to fit the EIS spectra, where CPEd refers to the high-frequency processes associated with the bulk aqueous phase and CPEp to those related to the interface between cellulose particles and water. Error bars represent the fitting uncertainty associated with the quantification of Rp obtained from the equivalent circuit analysis.

The adopted configuration enables a sensitive investigation of interfacial processes at the particle–electrolyte boundary, where variations in surface chemistry and charge-exchange kinetics are reflected in measurable changes in the impedance spectra [58]. The adopted methodology is consistent with recent studies demonstrating that modifications in surface functionality and interfacial charge transfer can be effectively detected by EIS [59]. The EIS characterizations are reported in Figure 7A, where the Nyquist plots for the aqueous suspensions containing 1 mg/mL of WC4, WC5, WC11, WC14, and WC20 are shown. As it is possible to see, a non-ideal semicircle can be observed from the plots, which could be typically described by a resistor and capacitor (RC) circuit in parallel. Importantly, the resistance is defined as polarization resistance (Rp), modeled as the apparent electrical resistance measured under an alternating current (AC) field when charges migrate and accumulate at water/particles, which derives from interfaces between materials having different electrical properties, in this case, cellulose and water. Importantly, such Rp is not related to the conventional electron transfer due to a Faradaic process, since no electron transport occurs between the particles and the electrode, whereas it models the charge migration resistance and the capacitor ability to store charge at the particle–liquid interfaces. To quantify the effect of grafting on the impedance characteristics, an equivalent circuit was adopted, which is reported in Figure 7B, which consists of a solution constant phase element, which takes into account the capacitive reactance of the dispersion (modeled with a constant phase element and defined as CPE_d_) in series with a parallel combination of an Rp and a constant phase element (CPE_p_), representing the non-ideal capacitive behavior to store charges at the particle–water interface. Figure 7B shows the corresponding plot reporting the variation in the Rp along with the P_G_ for the investigated samples. The samples were chosen considering a scale of low-to-high P_G_ values (from 27 to 93%). For each sample, the Rp values were obtained by fitting the experimental data to the equivalent circuit shown in the figure, which was employed to model the electrochemical behavior of the system and to extract quantitative parameters. Notably, the extracted Rp values display a decreasing trend with increasing P_G_: Rp drops from 14.9 kΩ at 27% P_G_ to about 7.5 kΩ at 61% P_G_, then reaches a plateau around 8.8 kΩ for higher P_G_ values.

This behavior can be interpreted considering the MWS model: at low grafting degrees (WC11), the contrast in permittivity and conductivity between cellulose and the acrylate chains produces relatively higher interfacial polarization. As functionalization increases, the acrylate chains more uniformly cover the particle surfaces, facilitating the redistribution of polarization and thereby decreasing Rp. For grafting degrees above approximately 60% (WC5, WC20), no further appreciable changes in Rp are observed. This stabilization can be reasonably attributed to the reorganization of the polymer chains at the interface, leading to a more ordered arrangement, and consequently, a relatively lower polarizability compared to intermediate grafting levels, such as WC4 and WC14 [60]. Additionally, the possible formation of a continuous dielectric layer at high grafting levels may further reduce the permittivity and conductivity contrast between phases, leading to a limited variation in Rp despite the increased functionalization [61]. Similar trends have been reported for other functionalized interfaces; in particular, activated carbon and MXene-based systems exhibit comparable effects, with Rp decreasing rapidly at low functionalization degrees and then stabilizing as surface coverage becomes dense [62,63].

In particular, in aqueous suspensions at lower concentrations (1 mg/mL, Figure 7A), the EIS spectra changes correlate with efficient interfacial polarization between the aqueous phase and the functionalized cellulose surface. Furthermore, measurements were also conducted at higher suspension concentrations, where the overall impedance increased (Figure S9A in the Supplementary Materials). This behavior is probably due to interparticle interactions increased by the suspension concentration, leading to the formation of a denser network of interfaces, where the polarization fields of neighboring particles overlap. This collective arrangement limits the effective development of local MWS polarization, resulting in a more resistive and capacitive dielectric response [64,65].

In the same context, Figure S9B reports the Bode plots of the samples, highlighting the frequency-dependent behavior of the impedance magnitude. A progressive decrease in impedance with an increasing functionalization degree is evident, particularly in the intermediate frequency range, indicating enhanced interfacial polarizability and an improved interfacial dielectric response at the particle–water interface.

The trend observed in the impedance data aligns closely with the adsorption performance of the same materials toward diesel fuel in water and seawater: increasing the grafting degree not only enhances the density of polar acrylate groups, which promotes interfacial polarization under an external field, but also introduces hydrophobic domains that modulate the structure and organization of interfacial water [16]. Together, these effects govern the dielectric polarization at the particle–water interface and modulate the organization of the interfacial water layer, which in turn influences the material’s ability to interact with and adsorb nonpolar or weakly polar species dispersed in the aqueous phase.

## 4. Conclusions

In conclusion, the goal of developing a bio-based fuel sorbent from agri-food waste was successfully met. Cellulose derived from citrus peel waste was used as a renewable and low-cost starting material to produce a copolymer with poly(methyl acrylate). The graft radical polymerization procedure was optimized, obtaining a high value of P_G_ (93% for material WC20) in a relatively simple manner. The successful functionalization of cellulose enabled the material to acquire effective fuel adsorption properties. The adsorption capacity of three selected materials—WC11, WC4, and WC20—was assessed for diesel and gasoline using a continuous-flow system, in oil/freshwater and in oil/seawater systems. The results showed that the cellulose gained adsorption capability only after functionalization with poly(methyl acrylate) chains, with performance increasing alongside the grafting percentage. Among the tested samples, the material with the highest P_G_ value (WC20) exhibited superior adsorption of both diesel and gasoline, confirming that adsorption performance correlates with the value of P_G_. Electrochemical impedance spectroscopy measurements realized on materials with increasing P_G_ revealed that both particle functionalization and suspension concentration influence the electrical response of the system. Specifically, EIS data indicate that higher grafting leads to a more uniform coverage of polymer chains on cellulose surfaces, improving the organization of interfacial water. In turn, this result agrees well with the fuel adsorption studies, meaning that grafting results in a greater interaction with hydrophobic systems, such as those of fuels, which are therefore more efficiently adsorbed at the particle interface.

The lower sorption capacity can be attributed to limited packing continuity, which reduces the contact time between the sorbent material and the fluids. Then, although the literature reports other cellulosic materials with higher adsorption capacity, this is the first example of cellulose derived from citrus peel waste used for fuel adsorption and in a real seawater sample. It is noteworthy that the material has no purchase cost, and recycling this waste avoids the high disposal costs for companies. For these reasons, the preliminary findings on this widely available, sustainable, and bio-based material pave the way for its most promising applications in the future. The materials could be further optimized for selective adsorption of different hydrocarbons or for integration into larger-scale water treatment systems. Additionally, combining cellulose-based sorbents with other functional polymers or nanomaterials may enhance adsorption efficiency and recyclability, broadening their environmental and industrial impact. Such developments could provide practical solutions for marine oil spill remediation and wastewater purification, while reinforcing the role of bio-based materials in sustainable technologies.

## Figures and Tables

**Table 1:** Preliminary study of graft copolymerization onto MCC and WC.

Entry ^1^	Product	Starting Material	Mercerization	P_G_ (%)
1	MCC1	MCC	No	-
2	MCC2	MCC	Yes	7.5
3	WC1	WC	No	1
4	WC2	WC	Yes	16

^1^ reaction condition: nitrogen atmosphere, degassed water (10 mL), methyl acrylate (1 mL), FAS (2.2 mg), KPS (75.4 mg), 80 °C, 24 h.

**Table 2:** Optimization of the reaction parameters to obtain maximum graft copolymerization onto mercerized cellulose derived from CPWs.

Entry ^a^	Product	Monomer (mol/L)	Solvent (mL)	FAS/KPS (mol:mol)	Time (min)	T (°C)	P_G_ (%)
1	WC3	0.16	100	1:1	120	55	47
2	WC4	0.20	100	1:1	120	55	56
3	WC5	0.24	100	1:1	120	55	86
4	WC6	0.28	100	1:1	120	55	60
5	WC7	0.32	100	1:1	120	55	62
6 ^b^	WC8	0.24	100	1:1	120	55	80
7 ^c^	WC9	0.24	100	1:1	120	55	2
8	WC10	0.24	50	1:1	120	55	10
9	WC11	0.24	125	1:1	120	55	27
10	WC12	0.24	100	1:0.5	120	55	5
11	WC13	0.24	100	1:0.3	120	55	4
12	WC14	0.24	100	0.3:1	120	55	61
13	WC15	0.24	100	0.5:1	120	55	51
14	WC16	0.24	100	1:1	60	55	12
15	WC17	0.24	100	1:1	90	55	74
16	WC18	0.24	100	1:1	150	55	55
17	WC19	0.24	100	1:1	120	45	3
18	WC20	0.24	100	1:1	120	65	93
19	WC21	0.24	100	1:1	120	75	81

^a^ reaction condition: open air; solvent: water; monomer: methyl acrylate; initiator: FAS/KPS; heating. ^b^ reaction conducted in an inert atmosphere (under nitrogen and by using degassed water). ^c^ reaction conducted without the soaking step.

**Table 3:** Oil adsorption capacity, S, with respect to different types of oils and different oil/water systems, measured in a continuous-flow system.

Entry	Sorption Capacity	MCC	MWC	WC11	WC4	WC20
1	S_0_ *	0.0204 g	0.0202 g	0.0199 g	0.0201 g	0.0203 g
2	S (diesel)	0.56 ± 0.04 g/g	nd	nd	2.38 ± 0.09 g/g	3.87 ± 0.05 g/g
3	S (gasoline)	0.49 ± 0.03 g/g	nd	nd	1.84 ± 0.08 g/g	2.79 ± 0.04 g/g
4	S (diesel/art. seawater)	nd	nd	nd	2.45 ± 0.1 g/g	3.95 ± 0.06 g/g
5	S (gasoline/art. seawater)	nd	nd	nd	1.92 ± 0.06 g/g	2.91 ± 0.05 g/g
6	S (diesel/seawater)	nd	nd	nd	2.61 ± 0.08 g/g	4.01 ± 0.05 g/g
7	S (gasoline/seawater)	nd	nd	nd	1.96 ± 0.09 g/g	2.95 ± 0.04 g/g

* S_0_ is the initial weight of the adsorbent material.

**Table 4:** Maximum oil sorption capacities at equilibrium, rate constants, and correlation coefficients evaluated at 25 °C.

WC20	Diesel		Gasoline	
q_e_(mg/mg) _experimental_	89.4		68.1	
Pseudo-first-order	R^2^	0.9371	R^2^	0.9227
	q_e_ (mg/mg)	49.83	q_e_ (mg/mg)	92.73
	k_1_	0.032	k_1_	0.037
Pseudo-second-order	R^2^	0.9994	R^2^	0.9937
	q_e_ (mg/mg)	92.71	q_e_ (mg/mg)	78.74
	k_2_	0.909	k_2_	0.022

**Table 5:** Isotherm parameters for the adsorption of diesel and gasoline.

WC20	Diesel		Gasoline	
Langmuir	R^2^	0.9994	R^2^	0.9996
	q_m_ (mg/mg)	5.24	q_m_ (mg/mg)	74.07
	K_L_	1.0135	K_L_	0.15
Freundlich	R^2^	0.7826	R^2^	0.9525
	n	53.76	n	31.06
	K_F_	6.83	K_F_	5.94

## Data Availability

The original contributions presented in this study are included in the article/Supplementary Materials. Further inquiries can be directed to the corresponding authors.

## Supplementary Materials

The following supporting information can be downloaded at https://www.mdpi.com/article/10.3390/polym18010082/s1, Figure S1: FTIR spectra of MCC and WC; Figure S2: FTIR spectra of WC and MWC; Figure S3: Pseudo-first-order graph of WC20 in diesel/water system; Figure S4: Pseudo-first-order graph of WC20 in gasoline/water system; Figure S5: Isotherm of WC20 in diesel/water system; Figure S6: Isotherm of WC20 in gasoline/water system; Figure S7: Freundlich model of WC20 in diesel/water system; Figure S8: Freundlich model of WC20 in gasoline/water system; Figure S9: Nyquist plots of aqueous suspensions (10 mg/mL) of WC11, WC4, WC5, WC14, and WC20 samples (A). Bode plots of aqueous suspensions (1 mg/mL) of the same samples (B); Figure S10: 1H NMR of WC20 in DMSO-d6; Figure S11: 1H NMR of PMA in DMSO-d6; Figure S12: Contact-angle measurements of gasoline and diesel drops on WC20; Figure S13: Contact-angle measurements of a water drop on WC20.
