# Supplementary material for: Functionalized Cellulose from Citrus Waste as a Sustainable Oil Adsorbent Material
Source: Polymers (Basel). 2025 Dec 27;18(1):82. doi: 10.3390/polym18010082 (PMC12787734; doi:10.3390/polym18010082)
Supplement: Supplementary file 1 [file polymers-18-00082-s001.zip › polymers-3990740-supplementary.pdf]

Supplementary Materials

Functionalized Cellulose from Citrus Waste as a Sustainable Oil Adsorbent Material

Loredana Maiuolo [1], Antonio Jiritano [1], Paola Costanzo [1,*], Federica Meringolo [1], Vincenzo Algieri [1], Giuseppe Arrabito [2], Giorgia Puleo [2] and Antonio De Nino [1]

1 Department of Chemistry and Chemical Technologies, University of Calabria, Via P. Bucci, Cubo 12C, 87036 Rende, CS, Italy; loredana.maiuolo@unical.it (L.M.); antonio.jiritano@unical.it (A.J.); federica.meringolo@unical.it (F.M.).
2 IRCCS NEUROMED—Istituto Neurologico Mediterraneo, Via Atinense 18, 86077 Pozzilli, IS, Italy; vincenzo.algieri@unical.it (V.A.)
3 Department of Physics and Chemistry-Emilio Segrè, University of Palermo, 90128 Palermo Italy; giuseppedomenico.arrabito@unipa.it (G.A.); giorgia.puleo01@unipa.it (G.P.)
* Correspondence: paola.costanzo@unical.it (P.C.)

	Page
**Figure S1.** FTIR spectra of MCC and WC	S2
**Figure S2.** FTIR spectra of WC and MWC	S2
**Figure S3.** Pseudo first-order graph of WC20 in diesel/water system	S3
**Figure S4.** Pseudo first-order graph of WC20 in gasoline/water system	S3
**Figure S5.** Isotherm of WC20 in diesel/water system	S4
**Figure S6.** Isotherm of WC20 in gasoline/water system	S4
**Figure S7.** Freundlich model of WC20 in diesel/water system	S5
**Figure S8.** Freundlich model of WC20 in gasoline/water system	S5
**Figure S9.** Nyquist plots of aqueous suspensions (10 mg/mL) of WC11, WC4, WC5, WC14, and WC20 samples (A). Bode plots of aqueous suspensions (1 mg/mL) of the same samples (B).	S6
**Figure S10.** ^{1}H NMR of WC20	S7
**Figure S11.** ^{1}H NMR of PMA	S8
**Figure S12.** Contact-angle measurements of a gasoline and diesel drop on WC20	S9
**Figure S13.** Contact-angle measurements of a water drop on WC20	S9

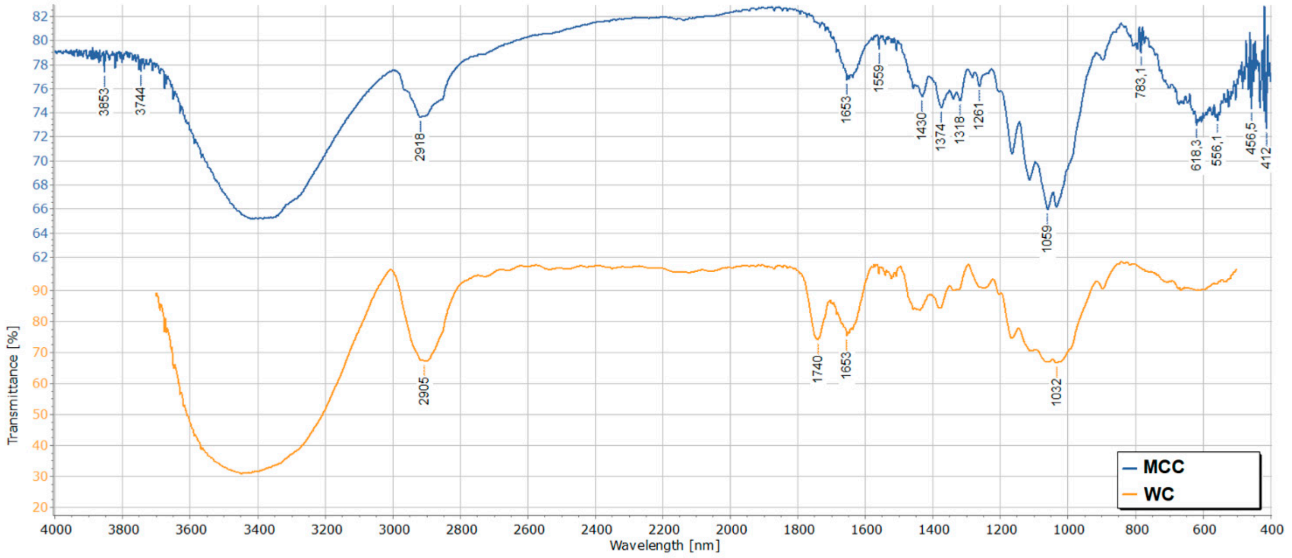

Figure S1. FTIR spectra of microcrystalline cellulose (MCC) at the top in blue and cellulose derived from Citrus Peel Wastes (WC) at the bottom in orange.

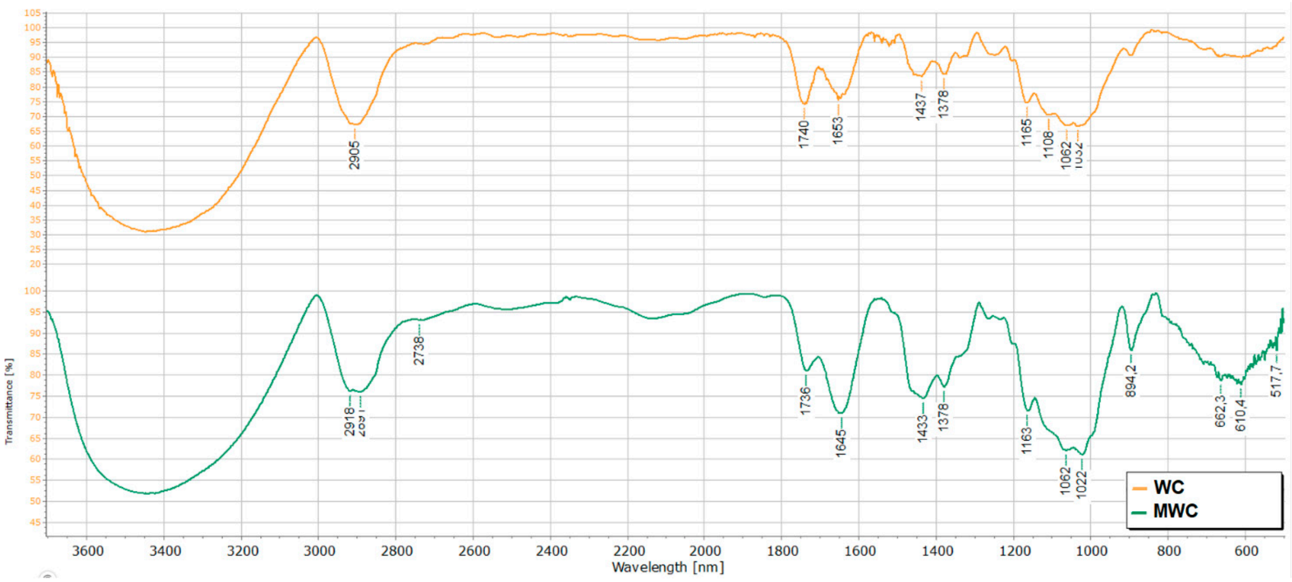

Figure S1. FTIR spectra of cellulose derived from Citrus Peel Wastes (WC) at the top in orange and mercerized cellulose derived from Citrus peel waste (MWC) at the bottom in green.

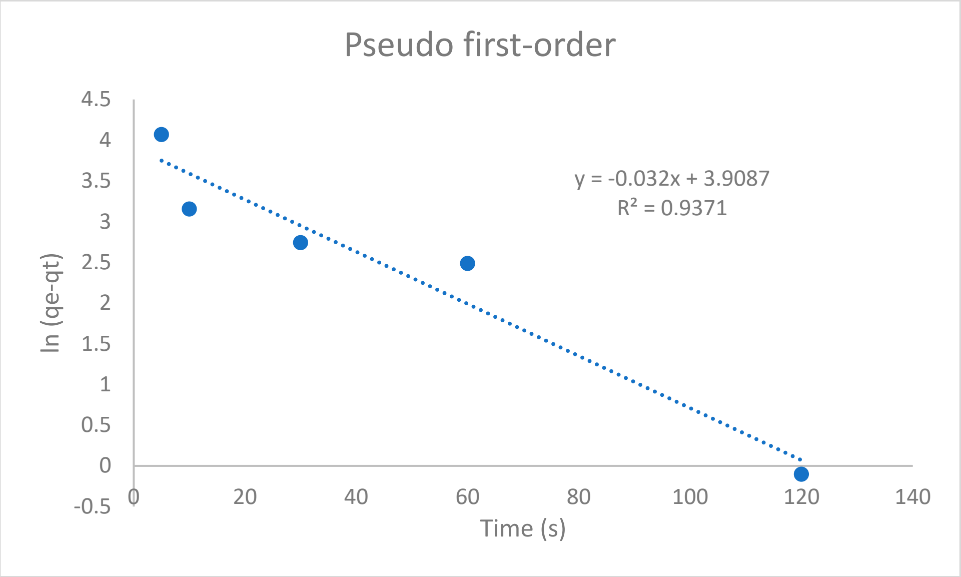

Figure S3. Pseudo first-order graph of WC20 in diesel/water system

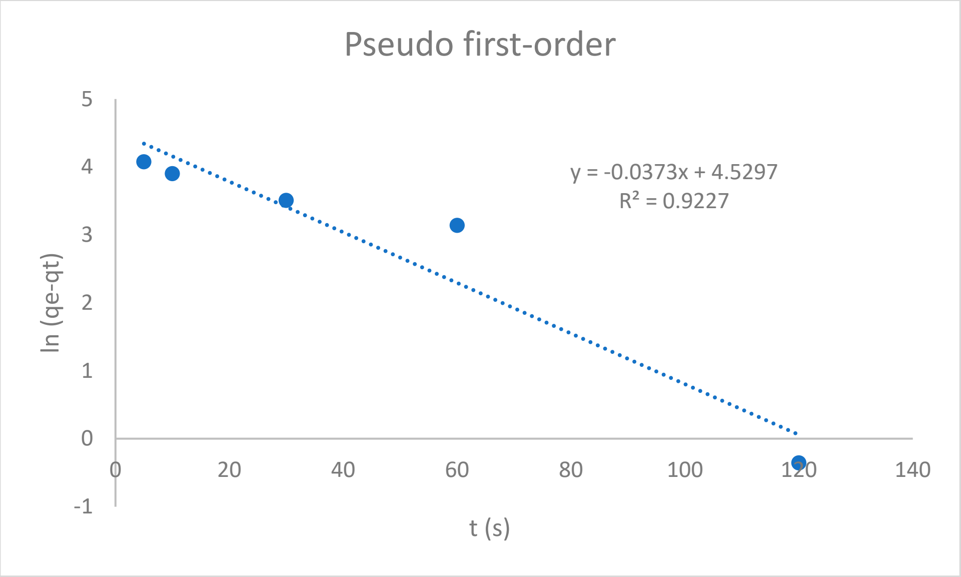

Figure S4. Pseudo first-order graph of WC20 in gasoline/water system

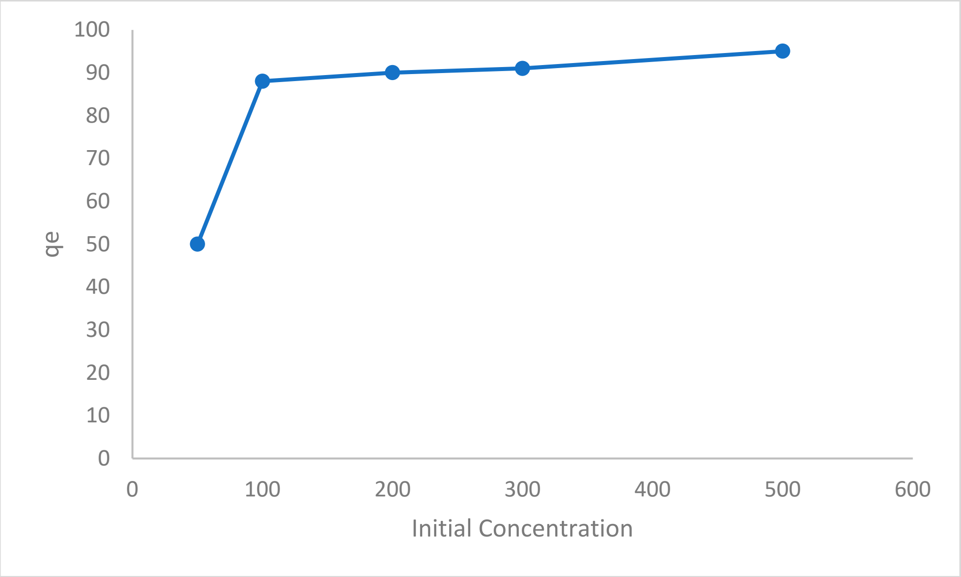

Figure S5. Isotherm of WC20 in diesel/water system

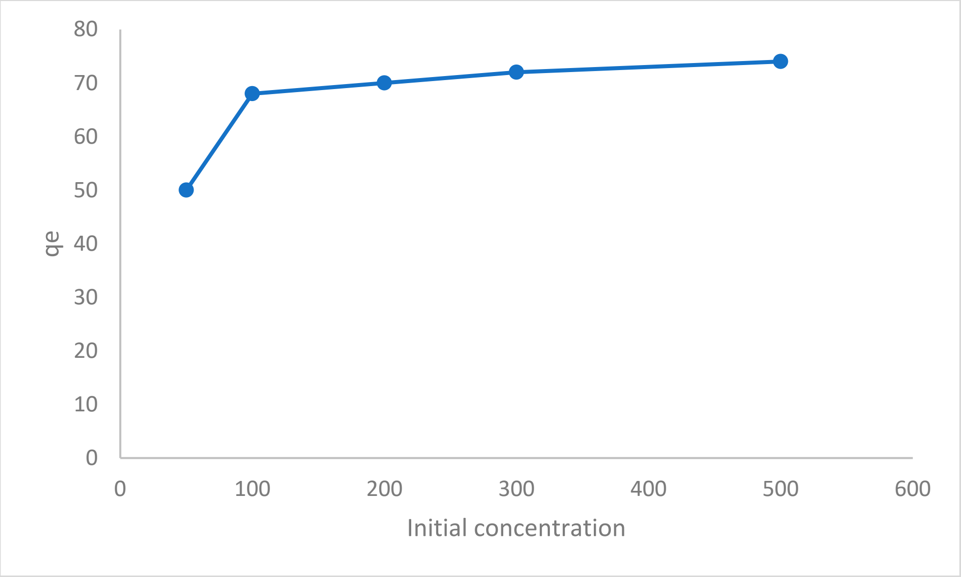

Figure S6. Isotherm of WC20 in gasoline/water system

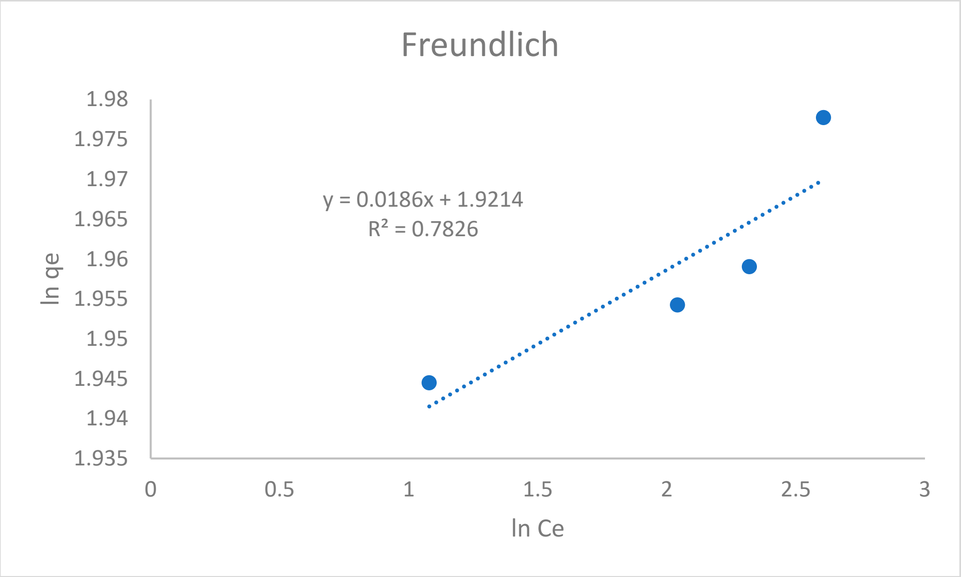

Figure S7. Freundlich model of WC20 in diesel/water system

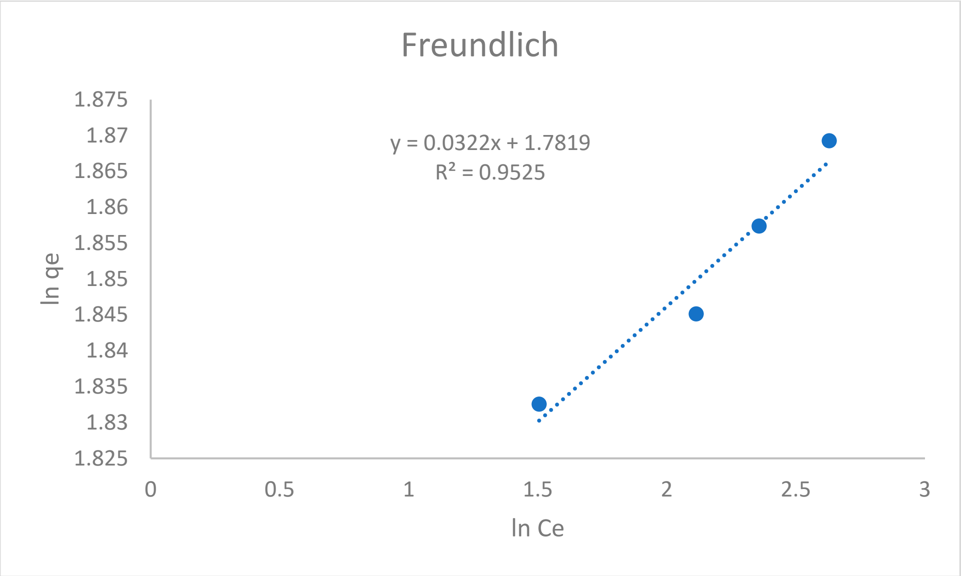

Figure S8. Freundlich model of WC20 in gasoline/water system

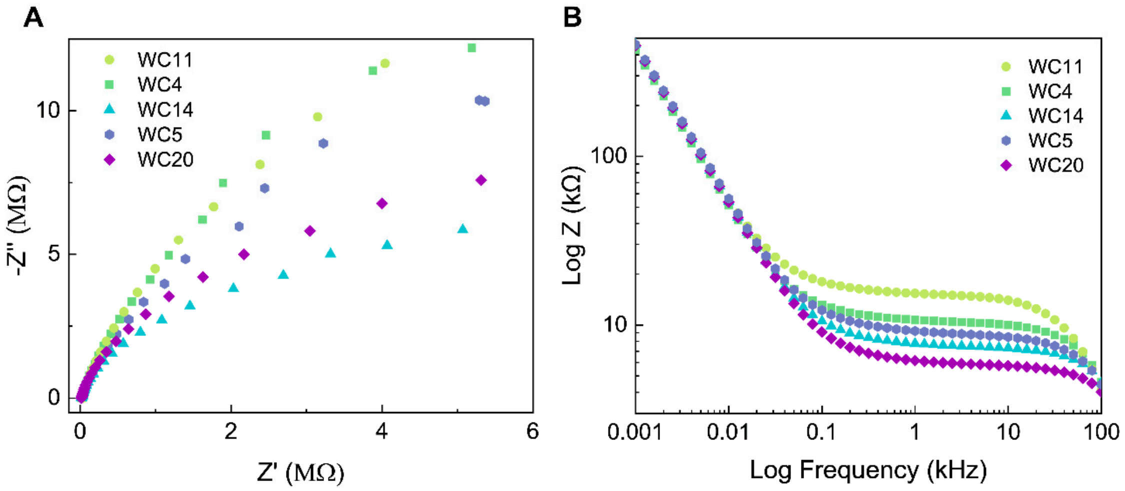

Figure S9. Nyquist plots of aqueous suspensions (10 mg/mL) of WC11, WC4, WC5, WC14, and WC20 samples (A). Bode plots of aqueous suspensions (1 mg/mL) of the same samples (B).

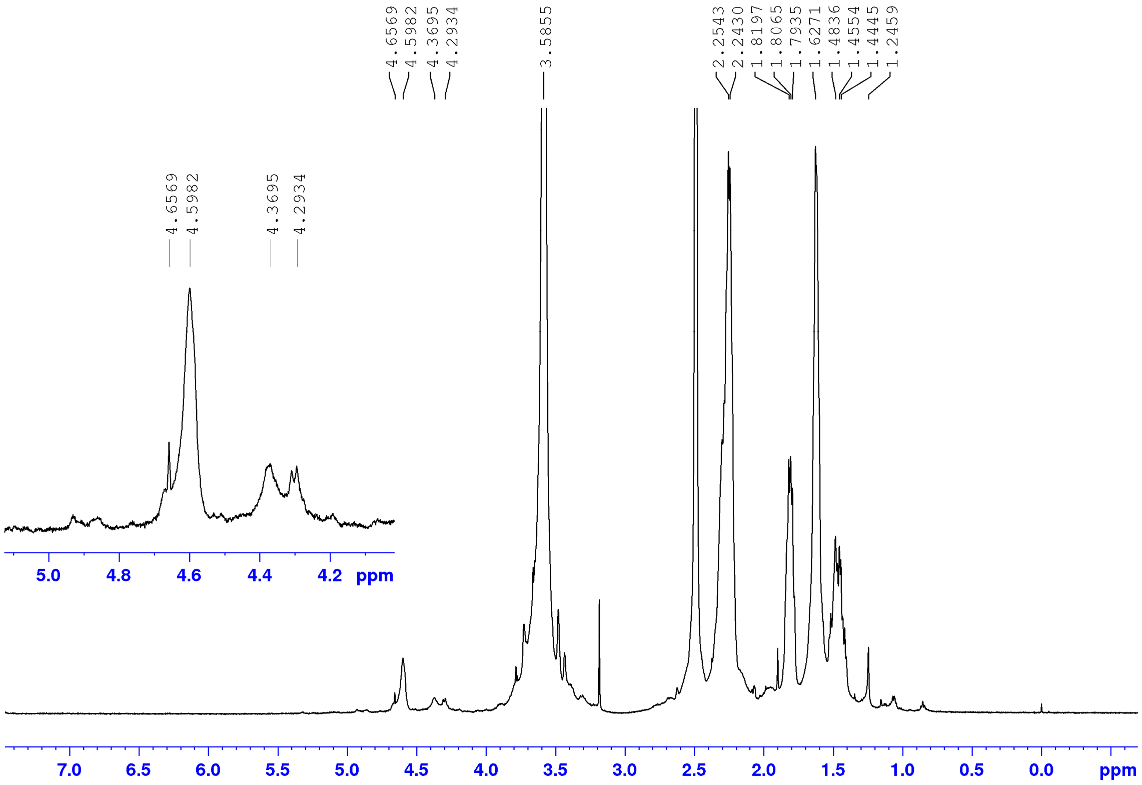

Figure S10. ^{1}H NMR spectra (in DMSO-d6) of material WC20 recorded at 37 °C.

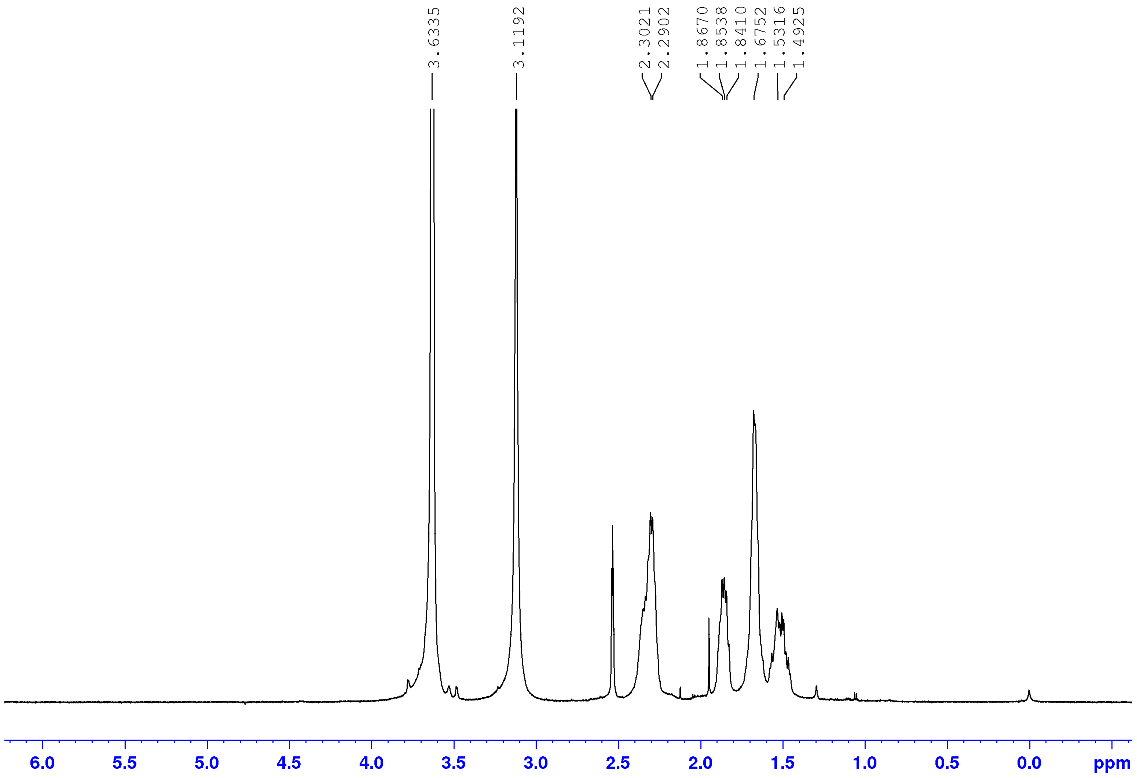

Figure S11. ^{1}H NMR spectra (in DMSO-d6) of PMA recorded at 37 °C.

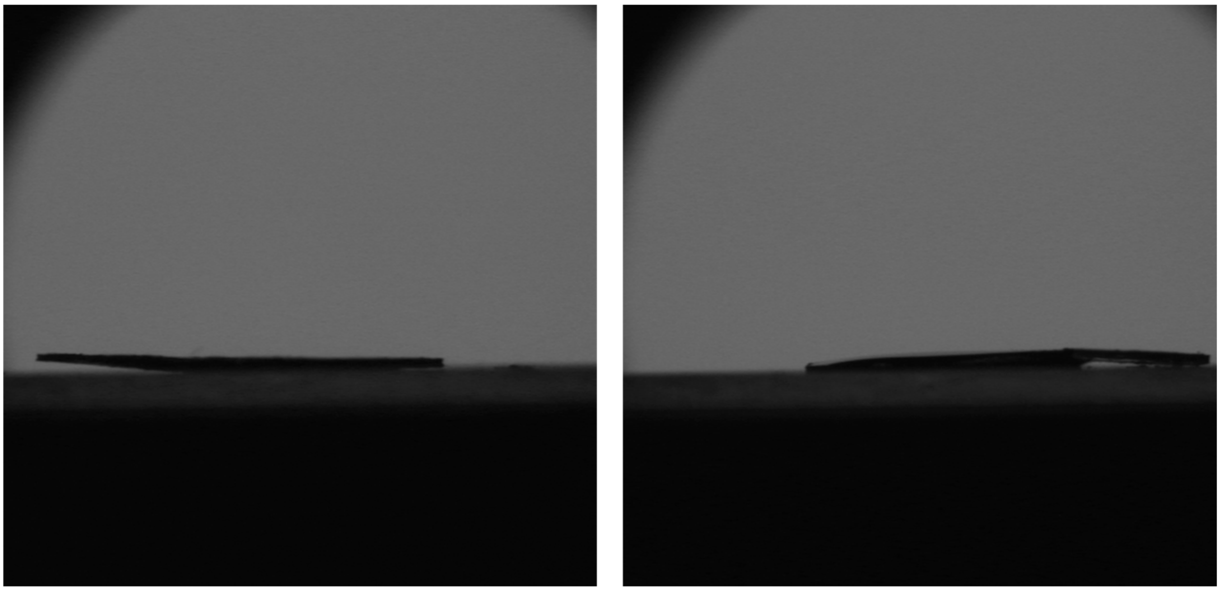

Figure S12. Image of the pellet surface (lateral view) taken after gasoline (left) or diesel (right) drop deposition

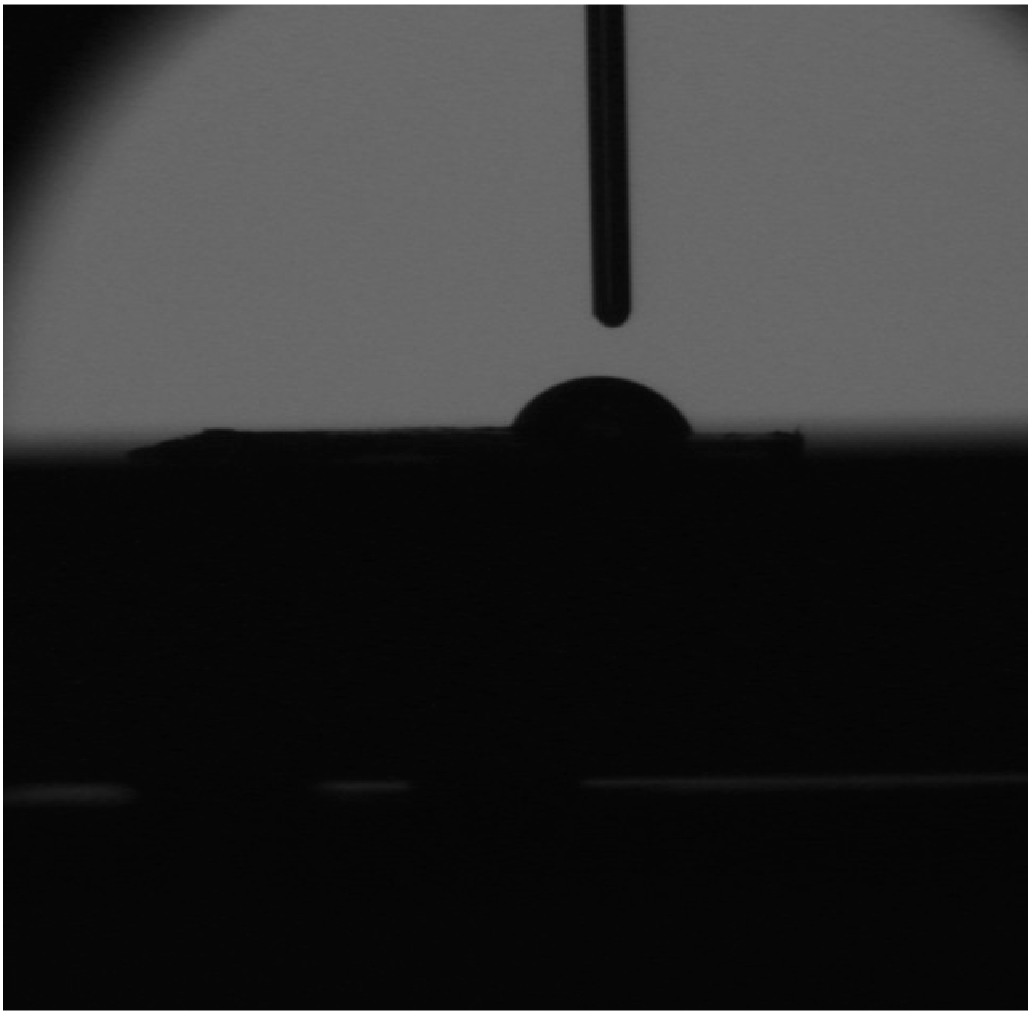

Figure S13. Image of the pellet surface (lateral view) taken after water deposition
